# Neonatal resuscitation using a supraglottic airway device for improved mortality and morbidity outcomes in a low-income country: study protocol for a randomized trial

**DOI:** 10.1186/s13063-019-3455-8

**Published:** 2019-07-19

**Authors:** Nicolas J. Pejovic, Susanna Myrnerts Höök, Josaphat Byamugisha, Tobias Alfvén, Clare Lubulwa, Francesco Cavallin, Jolly Nankunda, Hege Ersdal, Giulia Segafredo, Mats Blennow, Daniele Trevisanuto, Thorkild Tylleskär

**Affiliations:** 1 Centre for International Health, University of Bergen, Årstadveien 21, Box 7804, 5020 Bergen, Norway; 2 Sachs’ Children and Youth Hospital, Sjukhusbacken 10, 11883 Stockholm, Sweden; 3 Karolinska Institutet Department of Public Health Sciences, Tomtebodavägen 18A, 171 77 Stockholm, Sweden; 4 Mulago National Referral Hospital, Box 7272, Kampala, Uganda; 5 Department of Obstetrics and Gynaecology, College of Health Sciences, Makerere University, Box 7072, Kampala, Uganda; 6 Dept. of Woman and Child Health, 35128 Podova, Italy; 7 Department of Paediatrics and Child Health, College of Health Sciences, Makerere University, Box 7072, Kampala, Uganda; 8 Department of Anaesthesiology and Intensive Care, Stavanger University Hospital, Gerd-Ragna Bloch Thorsens gate 8, 4019 Stavanger, Norway; 9 Faculty of Health Sciences, University of Stavanger, Box 8600, 4036 Stavanger, Norway; 10 Operational Research Unit, Doctors with Africa Cuamm, Via San Francesco 126, Padova, Italy; 11 Department of Neonatal Medicine, Karolinska University Hospital, Eugeniavägen 3, 171 76 Stockholm, Sweden; 12 Karolinska Institutet Department of Clinical Science, Technology and Intervention, Alfred Nobels alle 8, 141 52 Huddinge, Sweden; 13 Department of Woman and Child Health, Padua University, Via Giustiniani, 3, 35128 Padua, Italy; 14 Centre for Intervention Science in Maternal and Child Health Centre for International Health, University of Bergen, Årstadveien 21, Box 7804, 5020 Bergen, Norway

**Keywords:** Global health, Low-income country, Laryngeal mask, Supraglottic airway device, Positive pressure ventilation, Newborn infant, Resuscitation, Neonatal mortality, Asphyxia, Asphyxia neonatorum, Intrapartum-related complications

## Abstract

**Background:**

Intrapartum-related death is the third leading cause of under-5 mortality. Effective ventilation during neonatal resuscitation has the potential to reduce 40% of these deaths. Face-mask ventilation performed by midwives is globally the most common method of resuscitating neonates. It requires considerable operator skills and continuous training because of its complexity. The i-gel^®^ is a cuffless supraglottic airway which is easy to insert and provides an efficient seal that prevents air leakage; it has the potential to enhance performance in neonatal resuscitation. A pilot study in Uganda demonstrated that midwives could safely resuscitate newborns with the i-gel^®^ after a short training session. The aim of the present trial is to investigate whether the use of a cuffless supraglottic airway device compared with face-mask ventilation during neonatal resuscitation can reduce mortality and morbidity in asphyxiated neonates.

**Methods:**

A randomized phase III open-label superiority controlled clinical trial will be conducted at Mulago Hospital, Kampala, Uganda, in asphyxiated neonates in the delivery units. Prior to the intervention, health staff performing resuscitation will receive training in accordance with the Helping Babies Breathe curriculum with a special module for training on supraglottic airway insertion. A total of 1150 to 1240 babies (depending on cluster size) that need positive pressure ventilation and that have an expected gestational age of more than 34 weeks and an expected birth weight of more than 2000 g will be ventilated by daily unmasked randomization with a supraglottic airway device (i-gel^®^) (intervention group) or with a face mask (control group). The primary outcome will be a composite outcome of 7-day mortality and admission to neonatal intensive care unit (NICU) with neonatal encephalopathy.

**Discussion:**

Although indications for the beneficial effect of a supraglottic airway device in the context of neonatal resuscitation exist, so far no large studies powered to assess mortality and morbidity have been carried out. We hypothesize that effective ventilation will be easier to achieve with a supraglottic airway device than with a face mask, decreasing early neonatal mortality and brain injury from neonatal encephalopathy. The findings of this trial will be important for low and middle-resource settings where the majority of intrapartum-related events occur.

**Trial registration:**

ClinicalTrials.gov. Identifier: NCT03133572. Registered April 28, 2017.

**Electronic supplementary material:**

The online version of this article (10.1186/s13063-019-3455-8) contains supplementary material, which is available to authorized users.

## Background

### Problem statement

Since 2015, after Millennium Development Goal number 4 (MDG-4), of globally reducing by two thirds the under-5 (years of age) mortality, was summarized, it has become evident that neonatal mortality does not decrease at the same pace as post-neonatal mortality [1].

Of the 140 million babies born in the world annually, 7–9 million will need resuscitation at birth. The latest estimates are that 662,000 deaths annually are caused by intrapartum-related events, commonly referred to as birth asphyxia, which is the third leading cause of under-5 mortality globally [2].

Key health indicators from Uganda in 2017 show that child (under-5) mortality decreased from 175 out of 1000 in 1990 to 53 out of 1000 in 2016 [3]. The rate of neonatal mortality, however, is estimated at 27 out of 1000 and remains unchanged despite the national roll-out of programs such as Helping Baby Breathe (HBB) [3, 4], a basic neonatal resuscitation curriculum for resource-limited settings aiming at improving skilled attendance at birth [5]. HBB implementation trials have demonstrated a reduction in fresh stillbirths and first-day neonatal mortality. However, recent studies in India, Kenya, and Nepal assessing long-term outcomes showed no change in overall 28-day neonatal mortality or perinatal mortality [6, 7].

Sustainable Development Goal number 3 (SDG-3) re-emphasizes the need of accelerating the reduction of neonatal mortality; each country should aim for a neonatal mortality below 12 out of 1000 live births by 2030. Achieving this goal will be possible only if we improve existing neonatal resuscitation programs [8]. All birth attendants, including physicians, midwives, and nurses, should have the knowledge and skills required to perform effective neonatal resuscitation [9]. Innovative tools that can strengthen existing strategies will have to be rapidly implemented if we are to reach the 12 out of 1000 target of neonatal death by 2030.

### Rationale

Providing positive pressure ventilation (PPV) is the single most important component of successful neonatal resuscitation [8, 9]. Yet the mortality of newborns needing face mask (FM) ventilation was as high as 10% in Tanzania [10].

Effective ventilation during neonatal resuscitation has the potential to reduce 40% of intrapartum-related deaths [11]. However, the delivery of proper tidal volume is a difficult technique to master. Mask leakage, airway blockage, and poor chest expansion have been reported during FM ventilation [12–14].

Ventilation is routinely initiated with FM followed by endotracheal intubation in case of FM ventilation failure or need for prolonged ventilatory support. Endotracheal intubation is the most difficult skill to master in neonatal resuscitation and performed only by experienced physicians [15]. The use of endotracheal tube (ETT) is not included in resuscitation guidelines aimed at low-resource settings [16].

The American Heart Association and the European Resuscitation Council guidelines have proposed the use of the laryngeal mask airway (LMA) to replace FM if ventilation is ineffective or as an alternative to ETT during resuscitation of the late-preterm and term infants (at least 34 weeks’ gestation or birth weight of more than 2000 g or both) if intubation is unsuccessful [17].

Several publications, including a recent Cochrane review [18, 19], have shown that the LMA allowed effective PPV in most of the treated patients (range of 95–99%) [20–24], reducing the need for intubation [25, 26]. In previous studies, an inflatable size 1 laryngeal mask was used [21, 23–27].

The i-gel^®^ (Intersurgical Ltd., Wokingham, Berkshire, UK) size 1 is a new model of cuffless supraglottic airway device that has recently been made available for newborns (2–5 kg). It is designed to provide an efficient seal to the larynx without the inflatable cuff used in the traditional LMA. Positioning is easy with a low risk of tissue compression or dislodgement [28–30]. All of these characteristics make the i-gel^®^ a potentially useful alternative to FM and ETT, especially in settings where the staff skills in performing PPV are insufficient [25–27]. A prospective observational study of 50 children demonstrated a success ratio of 100% for the insertion of the i-gel^®^. All devices were inserted on the first attempt. The study showed very few complications and concluded that it seems to be a safe and efficient device for pediatric airway management [31].

Task shifting the use of a cuffless supraglottic airway device to non-doctor or inexperienced health staff in resource-limited settings could be one way to improve outcome following newborn resuscitation. A manikin study in Uganda demonstrated that midwives could easily insert a cuffless supraglottic airway after brief on-the-job training: the i-gel^®^ was also more effective than FM in establishing PPV in the manikin. In 2015, a phase II randomized controlled trial (RCT) on the same site demonstrated that midwives could effectively and safely perform resuscitation in neonates with the i-gel^®^ [32, 33].

The effectiveness and safety of a supraglottic airway device compared with FM, as the primary interface for newborn resuscitation, are still identified as important knowledge gaps. The critical outcomes of mortality and indicators of brain damage also need to be assessed [34]. The proposed trial will follow the SPIRIT (Standard Protocol Items: Recommendations for Interventional Trials) guidelines [35] and provide evidence to determine whether use of a supraglottic airway device translates into better clinical outcomes and thus can be considered part of future guidelines for neonatal resuscitation in resource-limited settings (Additional file 1). The aim of the present trial will be to compare the effectiveness of two interfaces (i-gel^®^ versus FM) for administering PPV at birth in terms of 7-day mortality and neonatal encephalopathy.

## Methods/design

### Trial design

A randomized phase III open-label superiority controlled clinical trial will be conducted in neonates needing PPV at birth with two parallel groups (1:1 ratio): resuscitation with a supraglottic airway device (i-gel^®^) compared to FM (standard of care).

### Setting

This trial will be conducted in Uganda at the Delivery Unit and Operating Theatre of the Department of Obstetrics and Gynaecology at Mulago National Referral Hospital, Kampala, which has about 25,000 annual deliveries.

### Inclusion criteria

Inborn infants fulfilling the following inclusion criteria will be eligible to participate in the trial:

- Inborn baby (i.e., born in the hospital)
- Estimated gestational age of at least 34 weeks
- Estimated birth weight of at least 2000 g
- Need for PPV at birth (based on HBB algorithm)
- Parental consent.

### Exclusion criteria

- Major malformations (incompatible with sustained life or affecting the airways)
- Macerated stillbirth.

### Primary outcome measures

- A composite outcome of (a) 7-day mortality or (b) admission to neonatal intensive care unit (NICU) with neonatal encephalopathy (maximum Thompson score of 11 or above at day 1–5 during hospitalization) or both [36–38].

### Secondary outcome measures

- Safety of i-gel^®^ in the hands of lower cadre (non-doctor) birth attendants: adverse events (AEs) and serious adverse events (SAEs)
- Time to initiate PPV
- Heart rate at 0, 60, 90, 120, 180, 240, and 300 s
- Advanced resuscitation (chest compressions, intubation, and drug delivery), including intervention by supervising physician
- Early neonatal death (<7 days)
- Very early neonatal death (<24 h)
- Neonatal encephalopathy: admission to NICU with a Thompson score of 11 or above during day 1–5 during hospitalization
- Neonatal encephalopathy: admission to NICU with a Thompson score of 7 or above at day 1–5 during hospitalization
- Any hospital admission during the first 7 days of life.

### Procedures

#### Prior to interventions: training midwives

Two hundred members of the staff involved in neonatal resuscitation participated in a modified HBB (2nd edition) one-day course [5] during two weeks in November 2017. The course was held by two pediatricians familiar with the use of supraglottic airway devices and was facilitated by two or three local HBB instructors. It consisted of a review of the HBB action plan and practical hands-on skill stations. The HBB training includes simulation scenarios involving key procedures of the action plan (thermal loss prevention, stimulation, clinical assessment, airway management, etc.) and the use of the FM (Laerdal silicon resuscitator, Laerdal Medical, Stavanger, Norway). An additional module for training on the use of the i-gel^®^ (Intersurgical Ltd.) was added. A high-fidelity model (SimNewB Laerdal manikin, Laerdal Medical) was used to train the staff in the use of both devices (i-gel^®^ and FM). SimNewB provides realistic airways and good feedback with chest rise when effective PPV is provided. The participants learned the insertion technique recommended by the manufacturer that is the same in the manikin and in the neonate [26, 32]. A silicon lubricant (not needed in newborn infants because of oral secretions) facilitated the procedure. Three successful i-gel^®^ insertions in the manikin were required to partake in the study. FM ventilation was taught in accordance with the HBB curriculum using the NeoNatalie manikin (Laerdal Medical) and included advanced corrective measures. In case of failed FM ventilation, the participants were instructed to apply the following measures before considering the alternative airway device: reapplication of the mask, repositioning of the head, and increase of the inspiratory pressure. The use of suctioning was de-emphasized in accordance with the latest guidelines.

#### Recruitment and implementation

**Fig. 1:**
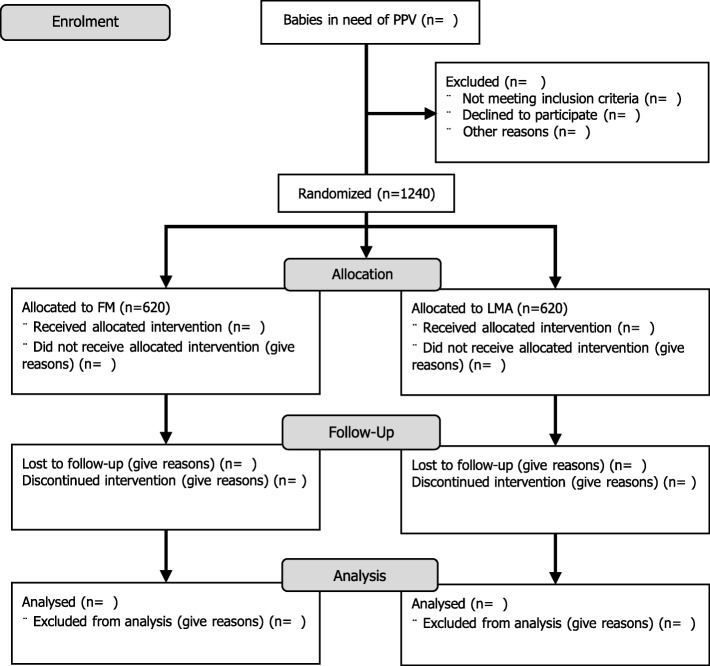
Trial profile (CONSORT flowchart)

Investigators and trained research assistants will participate in the enrollment of participants in accordance with the inclusion criteria (Fig. 1). Neonates will be recruited every day around the clock consecutively until sample size is reached. Data from babies will be used in the trial only after written parental consent is given. A senior investigator will be available at all times to discuss concerns raised by parents or clinicians during the course of the trial.

#### Tagging of newborns

All neonates enrolled in the trial and their mothers will be tagged with a trial bracelet with a unique trial ID number to facilitate matching and retrieval.

#### Randomization

Cluster randomization will be used, choosing day-by-day clusters. For practical reasons, individual randomization is not feasible, so all neonates enrolled in the same day (representing a cluster) will be randomly assigned to the same treatment. This approach randomly assigns daily groups of neonates rather than individual neonates, and neonates within any one day are likely to respond in a similar manner; hence, their data cannot be assumed to be independent. The clustering structure of the data was taken into account in sample size calculation and data analysis planning. A randomization list will be made by an independent statistician using block randomization with block sizes of 4–8. The allocation remains concealed until the actual trial day when the randomization envelope is opened by the surveillance officer on duty at 8 a.m. The midwives are informed at the beginning of each shift of the assigned treatment. The envelopes and assignment cards are discarded after use. The assigned procedure will then be performed until the next randomization. To provide proper PPV to the baby, the American Heart Association and European Resuscitation Council guidelines recommend switching to a supraglottic airway device if the resuscitator considers that the FM is failing [17]. We recommend the resuscitator to optimize the ventilation during 3 mins before considering switching ventilation option from FM to i-gel and vice versa, to keep contamination between arms low.

#### The intervention

Oral consent will be sought for all mothers admitted to the delivery unit, followed by deferred written informed consent as soon as practicable for mothers of babies eligible for the trial. HBB principles of the golden minute will be applied to all babies not crying at birth, including drying, stimulation, and assessment. A stopwatch will be started at the time of birth by a research assistant for all eligible participants. In the case of “baby is not breathing” after initial steps, the midwife will immediately (after cutting the cord) move the babies in need of PPV to the resuscitation area. Inflations will be administered with room air at a rate of 40 to 60/min with a 240-mL silicon self-inflating bag and a pop-off valve limit at 35 cm H_2_O (Laerdal Medical). Silicone, round-shaped FM (size 1, Laerdal Medical) or i-gel^®^ (size 1) will be available at each delivery. The duration of resuscitation will be defined as the time period from start of ventilation to the establishment of spontaneous breathing. Heart rate will be registered with a dry-electrode electrocardiogram monitor (NeoBeat Newborn Heart Meter, Laerdal Global Health, Stavanger Norway) featuring fast signal acquisition [39]. All babies with a 5-min APGAR (Appearance, Pulse, Grimace, Activity and Respiration) score of less than 7, respiratory distress, hypothermia (axillary temperature of less than 36.0 °C), or signs of encephalopathy will be transferred to the NICU. Resuscitation data, any contamination between arms, follow-up contact, and admission to the neonatal unit will be recorded by a research assistant. All interventions will be recorded on video to ensure quality assurance and data collection.

#### Management from supervising physician

Advanced resuscitation can be initiated in accordance with local hospital and International Liaison Committee on Resuscitation (ILCOR) guidelines [34], should a supervising physician be available. This can include use of alternative airways, including ETT, chest compressions, and drug administration.

#### Contamination between arms

Contamination between arms (switching to the alternative device) will be possible after 3 min of sustained PPV, should ventilation be deemed unsatisfactory. The alternative device will be accessible in an easily accessible box on the resuscitation table. This possible scenario will be practiced during the training. In all cases, a report specifying the reasons for switching to the alternative airway device will be filled out.

#### Masking

Health-care providers (midwives) performing resuscitation and the research assistant recording resuscitation data in the delivery ward cannot be masked to the allocation arm. However, the examiners assessing neonatal encephalopathy outcomes will be masked to the arm allocation. Outcome examiners will be exclusively working at the NICU, physically separated from where the resuscitations are performed. The arm allocation will not appear on the medical chart. Thus, arm allocation of admitted patients will not be identifiable by the outcome examiner. The independent data monitoring committee (IDMC) will have access to arm allocation when performing interim analysis and assessment of AEs/SAEs. The statistician who will perform data analysis will be masked to treatment allocation.

### Sample size

Considering our previous phase II trial, we estimate that a reduction of 25% of adverse outcomes may be possible. A sample size of 954 participants (477 per arm) is required to have a 90% chance of detecting, as significant at the 5% level, a decrease in the primary outcome measure from 40% in the standard-of-care arm to 30% in the supraglottic-airway arm. The sample size is increased to 1150 or 1240 because of the day-by-day cluster randomization, assuming an intra-class correlation of 0.10 and an average daily enrollment of three or four participants, respectively.

### Data collection and monitoring

#### Assessment and collection of outcomes

The primary outcome will be assessed in two parts. Mortality outcome will be collected daily at the NICU for admitted trial patients until day 7. Non-hospitalized participants will receive a scheduled appointment or phone call by a trial nurse with the mother at day 7 assessing the health of the baby. For all hospitalized participants, morbidity by neonatal encephalopathy will be assessed by a trial doctor masked to the arm allocation. This assessment will take place daytime on day 1, 2, 3, 4, and 5 or until discharge, using Thompson score (Table 1).

**Table 1:** Timeline of the trial

	Enrollment	Allocation	Admission neonatal intensive care unit		Follow-up
Time point	T-1	Day 0	Day 1	Day 2–5	Day 7
Enrollment					
Eligibility screen	×				
Prior oral consent	×				
Deferred consent			×		
Randomization		×			
Interventions					
Resuscitation		×			
Assessments					
Active monitoring of resuscitation		×			
Video recording		×			
Neurological status			×	×	
Mortality assessment			×	×	×

Data from the pre-coded case report form (CRF) will be entered into Open Data Kit (ODK) (https://opendatakit.org), an open-source suite of tools that helps researchers manage mobile data collection solutions. The data will be stored on an encrypted server and subsequently transferred to a statistical software package for analysis.

The CRFs will be pre-tested before the commencement of the trial. Data from the birth attendants’ questionnaire and the CRFs will be filled in by the birth attendants and will be continuously entered into ODK.

Videos will be recorded as a quality control. The neonatal resuscitation algorithm will be put in place to ensure that all interventions are standardized. A proper light source is needed on the table. Headlamps will act as backup in case of a power shortage at night.

#### Independent data monitoring committee

An IDMC consisting of four members—a statistician, an obstetrician, and two pediatricians—was appointed. They are operating in accordance with the IDMC charter which is developed with the members.

The timing of the interim analysis will be carried out by the IDMC. It will be planned when about half of the events have occurred, following the DAMOCLES (Data Monitoring Committees: Lessons, Ethics, Statistics) group recommendations [40].

The IDMC will ensure that the trial protocol was followed and control the adequacy of enrollment and randomization. The interim data will also assess quality standards and adherence to ethical requirements.

The interim analysis will be performed by the IDMC statistician unmasked to the treatment allocation. Based on this, the IDMC will make recommendations on the continuation of the trial and its modifications or decide on potential termination in case of harm.

### Statistical analysis

A detailed statistical analysis plan—based on the principles in this section—will be developed before the statistical analysis of the trial. Data analysis will be performed by using the statistical software packages Stata, SAS, and R. All tests will be two-sided, and a *P* value of less than 0.05 will be considered statistically significant. Missing data will be considered, and appropriate imputations will be discussed and performed when appropriate. Statistical analysis will include an unadjusted analysis followed by an adjusted analysis. The primary outcome will be compared between the two treatment arms by using the chi-squared test. The secondary outcomes will be compared by using the chi-squared test or Fisher’s test (categorical outcomes) and using the Student’s *t* test or Mann–Whitney test (continuous outcomes). Mixed-effect regression models will be estimated to evaluate the effect of the treatment on binary outcomes, adjusting for clusters (random effect) and clinically relevant confounders. Data analyses will be performed on an intention-to-treat (ITT) basis. However, since the trial is prone to some contamination (i.e., the person resuscitating may decide to shift to the other device) which can be limited by appropriate training but not entirely prevented, a per-protocol analysis and a contamination-adjusted ITT analysis will also be performed. These results will be considered along with the primary ITT analysis when drawing the conclusions of the trial. Subgroup analyses—per treatment center, time of the day (i.e., day/night), and per birth mode—will be carried out with exploratory purpose.

### Safety

Resuscitations will be continuously monitored by video and observed by the attending midwife or physician and the researcher assistant in order to detect AEs and SAEs. Safety measures will include monitoring of SAEs and detection of unexpected changes in incidence of common neonatal complications. The AEs will be managed by the attending hospital physician/midwife/researcher and followed until resolution or until a stable clinical end-point is reached by the clinician responsible for the care of the recruited patient.

If there is a reasonable suspected causal relationship with the intervention, SAEs will be reported to the Mulago Research and Ethics Committee (MREC) to guarantee the safety of the participants. Any suspected unexpected serious adverse reactions (SUSARs) with or without a reasonably plausible causal relationship with use of the supraglottic airway will also be reported to the MREC.

### Ethical considerations

The protocol was approved by the institutional review board of Mulago National Referral Hospital, Uganda; the Uganda National Council of Science and Technology; the Director General from the Ministry of Health, Uganda (MREC 1168); and the Regional Committee for Medical and Health Research Ethics (REK South East reference number 2017/989) in Norway.

Extensive discussions with clinical experts and members of the ethical board were necessary to solve the problem of obtaining consent without delaying the intervention. A two-tier procedure for consent will be implement in this trial because it involves unexpected care of critically ill newborns. All mothers entering the labor ward irrespective of whether their baby is suspected of filling inclusion criteria will receive brief information of the trial after which oral consent will be sought. Mothers whose infants are found eligible at birth will be approached for full written deferred consent for continuing participation. All information, including informed consent and the material used in the trial, will be translated in English and Luganda in a clearly understandable form. A senior investigator will be available to discuss any additional questions regarding the trial.

### Sustainability and scalability

A simplified neonatal resuscitation program that can reduce neonatal deaths due to perinatal asphyxia is the highest newborn global health research priority beyond 2015 [41]. This trial will try to demonstrate the first phase of scalability of an innovative approach to newborn resuscitation.

The training module for supraglottic airway use can easily be integrated to current neonatal resuscitation programs [33]. The cost-effectiveness of a supraglottic airway in a low-resource setting needs to be assessed. Such an investment can be justified only if there is a substantial difference between the supraglottic airway and FM. We estimate that a 25% reduction in adverse outcomes is a clinically significant difference large enough to have policy implications. A reusable cuffless device is already available but is still cost-prohibitive [29], so it will be crucial to explore how the unit cost can be reduced. A historical parallel could be the substantial drop in the cost of anti-retroviral therapy against HIV over the last decades [42], allowing scale-up of treatment to a level that previously seemed impossible in low-resource settings.

## Discussion

Newborn resuscitation training and simulation-based curriculum show mixed results in relation to their impact on newborn mortality [3, 4] and their effect on neurological morbidity remains unknown [43]. Further improvement of neonatal resuscitation performance is crucial.

This large trial is the first to assess the impact on mortality/morbidity of the use of a supraglottic airway device during neonatal resuscitation. It is powered to 90% and designed to add evidence lacking in this field. To the best of our knowledge, only four RCTs comparing LMA or supraglottic airway to FM ventilation including 636 patients have previously been published [18, 19, 25, 26, 32]. They have focused mainly on vital sign outcomes or successful resuscitation [34]. Safety and long-term outcomes remain important knowledge gaps. This task-shifting intervention involves midwives as they are the frontline health workers in many settings where newborn mortality is high. The burden of disease from intrapartum-related events can be reduced if simple and robust technologies for newborn resuscitation can be identified [44].

The trial will also monitor neonatal outcome data until day 7. We hypothesize that effective ventilation will be easier to perform with the supraglottic airway device and significantly decrease early neonatal mortality and brain damage from neonatal encephalopathy. Results from this large trial will contribute to provide evidence that can help define best practice advice for future guidelines.

### Trial status

The trial started recruiting participants on May 8, 2018.

## Additional file

Additional file 1: SPIRIT (Standard Protocol Items: Recommendations for Interventional Trials) guidelines. (DOCX 63 kb)

## Data Availability

Not applicable.

## Abbreviations

AE: Adverse event
CRF: Case report form
ETT: Endotracheal tube
FM: Face mask
HBB: Helping Babies Breathe
IDMC: Independent data monitoring committee
ITT: Intention-to-treat
LMA: Laryngeal mask airway
MREC: Mulago Research and Ethics Committee
NICU: Neonatal intensive care unit
ODK: Open data kit
PPV: Positive pressure ventilation
RCT: Randomized controlled trial
SAE: Serious adverse event
